# Crosstalk of extracellular vesicles in maternal-fetal interaction

**DOI:** 10.3389/fcell.2026.1884001

**Published:** 2026-08-10

**Authors:** Chen Zhou, Zhiqian Xu, Xiangbin You, Qiankun Wang, Xiaoxia Li, Youbing Yang

**Affiliations:** College of Animal Science and Technology, Henan University of Science and Technology, Luoyang, China

**Keywords:** extracellular vesicles, fetal growth restriction, gestational diabetes mellitus, maternal-fetal interface, placenta, preeclampsia, preterm birth, recurrent implantation failure

## Abstract

Extracellular vesicles (EVs) have emerged as key mediators of intercellular communication at the maternal–fetal interface and play indispensable roles in the establishment and maintenance of pregnancy. This review provides a mechanistic perspective on how EV-mediated signaling coordinates physiological maternal–fetal communication and how disruption of these signaling networks contributes to pregnancy-related disorders. We summarize current evidence demonstrating that EVs regulate multiple complementary biological processes, including maternal immune tolerance, trophoblast adhesion and invasion, placental angiogenesis and vascular remodeling, epithelial–stromal crosstalk, and metabolic and inflammatory adaptation, thereby maintaining maternal–fetal homeostasis. We further discuss how dysregulated EV biogenesis, cargo composition, and intercellular communication contribute to the pathogenesis of preeclampsia, gestational diabetes mellitus, preterm birth, recurrent implantation failure, and fetal growth restriction. In addition, the potential applications of EV-associated molecules as non-invasive biomarkers and the emerging use of engineered EVs for targeted therapeutic delivery are critically evaluated. Particular emphasis is placed on current challenges that limit clinical translation, including methodological heterogeneity, lack of standardized isolation and characterization protocols, insufficient biomarker validation, and pregnancy-specific safety considerations. Overall, a deeper understanding of EV-mediated maternal–fetal communication, together with methodological standardization and rigorous clinical validation, will facilitate the development of reliable EV-based diagnostic and therapeutic strategies for pregnancy-related disorders.

## 1 Introduction

Extracellular vesicles (EVs) are membrane-enclosed nanoparticles actively secreted by almost all cell types and typically range from approximately 30–1000 nm in diameter (Huang et al., 2026). They are widely distributed in biological fluids, including blood, urine, amniotic fluid, and breast milk, and function as important mediators of intercellular communication through the transfer of proteins, lipids, DNA, mRNAs, microRNAs (miRNAs), and other bioactive molecules (Soltanmohammadi et al., 2025; Yanez-Mo et al., 2015). Owing to their ability to regulate cellular signaling, immune responses, metabolic homeostasis, and gene expression, EVs have become an important focus of biomedical research over the past decade (Li et al., 2026).

Pregnancy represents a highly coordinated biological process that requires continuous communication between maternal and fetal tissues to support embryo implantation, placental development, immune adaptation, and fetal growth (Yu et al., 2022). At the maternal–fetal interface, EVs have emerged as critical mediators of this bidirectional communication network. EVs released from maternal tissues, trophoblasts, embryos, and placental cells deliver diverse molecular cargoes to recipient cells, thereby regulating maternal immune tolerance, trophoblast adhesion and invasion, angiogenesis, tissue remodeling, metabolic adaptation, and inflammatory homeostasis (Kaminski et al., 2019; Sun et al., 2025). Through these coordinated signaling events, EVs contribute to the establishment and maintenance of a physiological microenvironment that is essential for successful pregnancy.

Accumulating evidence further indicates that EV-mediated communication is highly dynamic throughout gestation. Both the abundance and molecular composition of EVs change in response to gestational stage, cellular origin, and physiological conditions (Hu et al., 2022; Nakamura et al., 2020). Under pathological conditions, alterations in EV biogenesis, cargo composition, and biological activity have been implicated in the development of pregnancy-related disorders, including preeclampsia (PE), gestational diabetes mellitus (GDM), preterm birth (PTB), recurrent implantation failure (RIF), and fetal growth restriction (FGR) (Popova et al., 2024; Sun et al., 2025; Zheng et al., 2025). For example, placental-derived EVs from women with PE exhibit altered miRNA and protein profiles that are associated with endothelial dysfunction and systemic inflammation (Aharon et al., 2023; Gebara et al., 2021). Similarly, dysregulated EV signaling has been linked to abnormal placental angiogenesis, impaired trophoblast function, metabolic disturbances, and excessive inflammatory responses in other pregnancy complications (Lin et al., 2026). These findings suggest that disruption of physiological EV-mediated communication represents a common mechanism contributing to pathological pregnancy.

In addition to their biological functions, EVs have attracted increasing interest as potential clinical tools. Because EVs are readily detectable in maternal circulation from early pregnancy and exhibit remarkable molecular stability, EV-associated proteins, nucleic acids, and lipids have been proposed as promising candidates for non-invasive biomarker discovery (Primorac et al., 2026; Simon et al., 2018). Furthermore, their intrinsic biocompatibility and natural targeting capacity make EVs attractive vehicles for therapeutic cargo delivery (Sun et al., 2023). Nevertheless, despite these encouraging advances, most reported EV biomarkers remain at the discovery stage, and the clinical translation of EV-based therapeutics is still limited by methodological heterogeneity, insufficient validation, and pregnancy-specific safety considerations.

Although previous reviews have separately summarized placental EVs, embryo–maternal communication, EV biomarkers, or the involvement of EVs in individual pregnancy-related disorders, these topics have largely been discussed in isolation. Consequently, the mechanistic links connecting physiological EV-mediated maternal–fetal communication with pathological EV dysregulation have not been comprehensively synthesized. In particular, the dynamic interplay among EV origin, cargo composition, recipient-cell specificity, signaling pathways, and disease phenotypes throughout pregnancy has yet to be integrated into a unified conceptual framework. Addressing this knowledge gap is essential for understanding how physiological EV-mediated signaling maintains pregnancy homeostasis and how its dysregulation contributes to the development of pregnancy-related disorders.

This article is presented as a narrative review. The literature was primarily retrieved from PubMed, Web of Science, and Scopus using combinations of the keywords “extracellular vesicles”, “exosomes”, “pregnancy”, “maternal–fetal interface”, “placenta”, “embryo implantation”, “preeclampsia”, “gestational diabetes mellitus”, “preterm birth”, “recurrent implantation failure”, and “fetal growth restriction”. The search mainly focused on peer-reviewed articles published between January 2010 and March 2026, while seminal earlier studies were included where appropriate to provide important biological context. Original research articles and authoritative review papers relevant to EV biology, maternal–fetal communication, pregnancy-related diseases, and clinical translation were preferentially included, whereas studies unrelated to pregnancy or lacking sufficient experimental evidence were excluded. Rather than providing a systematic or scoping assessment of all available evidence, this narrative review aims to provide an integrated mechanistic synthesis of current knowledge. Specifically, we summarize the biogenesis and biological characteristics of EVs, discuss how EV-mediated signaling regulates maternal immune tolerance, trophoblast adhesion and invasion, angiogenesis, epithelial–stromal crosstalk, and metabolic and inflammatory adaptation during normal pregnancy, and further examine how disruption of these physiological communication networks contributes to pregnancy-related disorders. Finally, we critically evaluate the current evidence supporting EV-associated biomarkers and therapeutic strategies, discuss the methodological and translational challenges that limit their clinical implementation, and highlight future research priorities for the development of EV-based diagnostic and therapeutic approaches in reproductive medicine.

## 2 Biogenesis and characteristics of EVs

EVs are membrane-bound particles secreted by nearly all cell types, playing a significant role in intercellular communication (Lee and Kim, 2022). The biogenesis of EVs primarily occurs through two pathways: exosomes and microvesicles (Wang Z. et al., 2024). Exosomes are generated within the endosomal system, where early endosomes progressively mature into multivesicular bodies (MVBs) that encapsulate cytoplasmic contents. These MVBs eventually fuse with the plasma membrane, releasing exosomes into the extracellular space (Arya et al., 2024). In contrast, microvesicles form through direct outward budding of the plasma membrane, a process regulated by cytoskeletal rearrangements and changes in membrane lipid composition (Chatterjee et al., 2024; Sedgwick and D'Souza-Schorey, 2018). Additionally, apoptotic bodies, which share structural similarities with EVs, are produced during programmed cell death and are typically larger in size (Kakarla et al., 2020).

In accordance with the Minimal Information for Studies of Extracellular Vesicles (MISEV) recommendations, the term “EVs” is preferentially used throughout this review unless the biogenetic origin of a vesicle population has been experimentally demonstrated (Welsh et al., 2024). Because many commonly used isolation methods recover heterogeneous vesicle populations, classification based solely on particle size or isolation procedures should be interpreted with caution. Therefore, the designation “exosomes” is used only when evidence supporting endosomal biogenesis is available, whereas the broader term “EVs” is adopted in other contexts.

**FIGURE 1:**
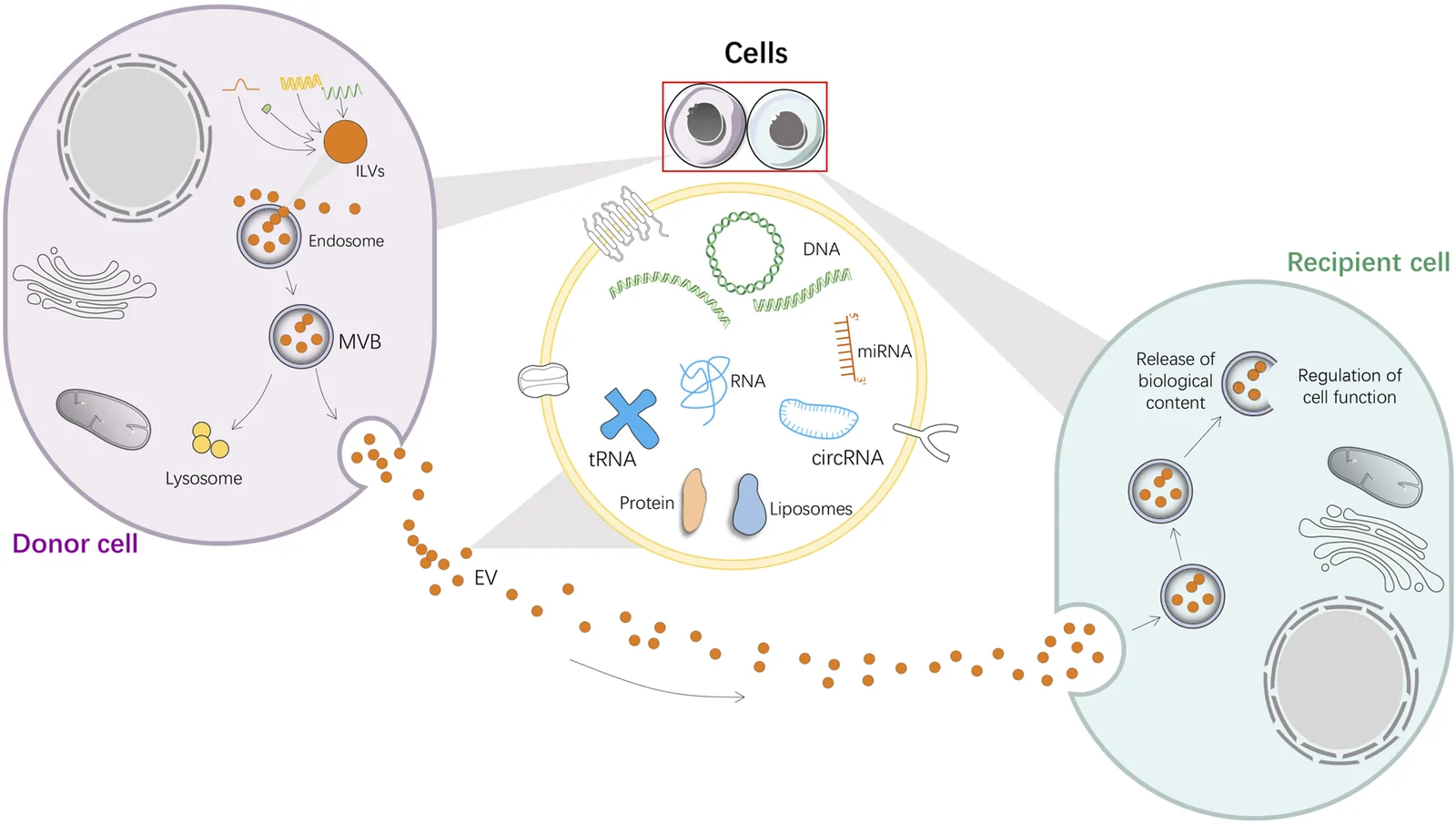
EVs biogenesis and intercellular communication. EVs arise via plasma membrane budding or endosomal pathways and include microvesicles and small EVs. They carry proteins, lipids, and RNAs, and are internalized by recipient cells through endocytosis, phagocytosis, or membrane fusion.

The characteristics of EVs depend on their cell of origin and the physiological state at the time of release. Typically, EVs range in size from 30 to 1000 nm, with exosomes being the smallest (30–150 nm), microvesicles being intermediate (100–1000 nm), and apoptotic bodies being the largest (>1000 nm) (Chitti et al., 2024). EVs carry a variety of bioactive substances, including proteins, lipids, and nucleic acids such as mRNA, miRNA, and circRNA (Payandeh et al., 2024). These contents reflect the physiological state of the originating cell and can be selectively packaged into EVs, influencing gene expression, signaling pathways, and functional responses in recipient cells (Yanez-Mo et al., 2015). The biogenesis of EVs, their molecular cargoes, and the major mechanisms of EV uptake by recipient cells are summarized in Figure 1. EVs possess unique surface markers that facilitate their recognition and targeting functions. Common exosomal markers include CD63, CD9, and CD81, which are tetraspanin proteins involved in vesicle formation and cargo selection. The lipid bilayer structure of EVs protects the encapsulated molecules, allowing bioactive cargo to be delivered intact to target cells, and even enabling long-distance transport within the body (Kumar et al., 2024).

Based on these characteristics, EVs are widely recognized as important mediators of intercellular communication, particularly in complex biological environments such as the maternal-fetal interface, where their significance is increasingly highlighted. EVs play a crucial role in maintaining homeostasis during pregnancy by transmitting genetic material, modulating immune responses, and regulating cellular behaviors (Tekkatte et al., 2023).

## 3 EV-mediated maternal–fetal communication during normal pregnancy

**FIGURE 2:**
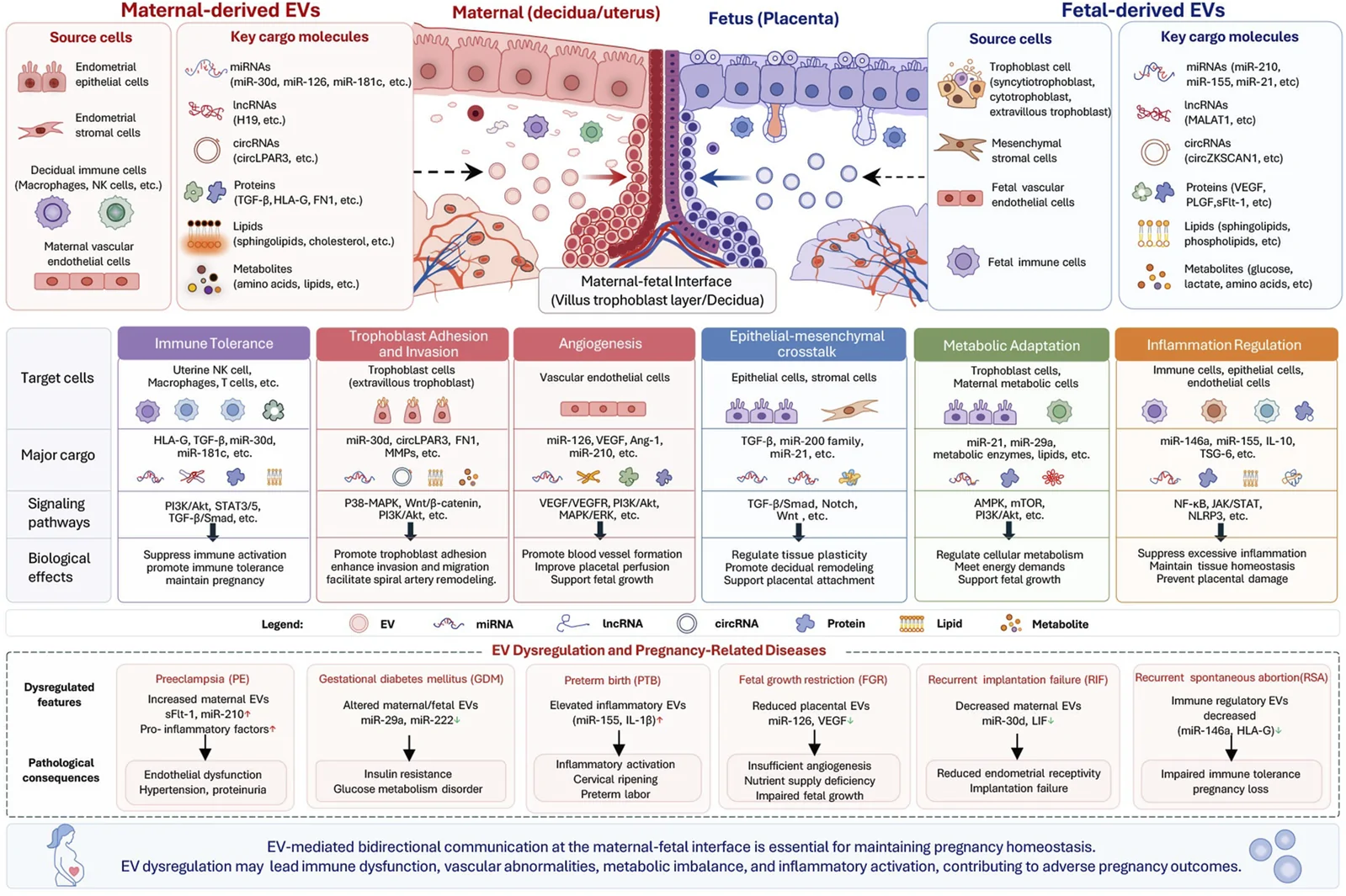
EV-mediated communication network at the maternal–fetal interface during normal pregnancy and pregnancy-related disorders.

EV-mediated communication forms a highly coordinated regulatory network at the maternal–fetal interface. During normal pregnancy, EVs derived from maternal tissues, trophoblasts, placental cells, and fetal tissues orchestrate immune tolerance, trophoblast invasion, angiogenesis, epithelial–stromal communication, and metabolic adaptation. Dysregulation of these signaling pathways contributes to the development of multiple pregnancy-related disorders. An overview of these physiological and pathological EV-mediated communication networks is presented in Figure 2.

### 3.1 EVs regulate maternal immune tolerance

Establishment of maternal immune tolerance is one of the earliest and most critical events during pregnancy. Unlike conventional allogeneic tissues, the semi-allogeneic fetus must evade maternal immune rejection while preserving sufficient immune surveillance against pathogens (Wang J. et al., 2024). This delicate balance is achieved through coordinated communication between trophoblasts and maternal immune cells at the maternal–fetal interface. Increasing evidence suggests that EVs function as central mediators of this communication by transferring immunomodulatory proteins, lipids, and regulatory RNAs that collectively suppress excessive maternal immune activation and promote fetal tolerance (Godakumara et al., 2022).

Among the immunoregulatory molecules associated with placental EVs, human leukocyte antigen-G (HLA-G) is one of the best characterized. HLA-G is a non-classical major histocompatibility complex class I molecule predominantly expressed by extravillous trophoblasts and can also be detected on trophoblast-derived EVs (Gu et al., 2025). EV-associated HLA-G interacts with inhibitory receptors, including immunoglobulin-like transcript 2 (ILT2) and ILT4, expressed on natural killer (NK) cells, dendritic cells, macrophages, and T lymphocytes, thereby suppressing NK-cell cytotoxicity, inhibiting antigen presentation, promoting regulatory T-cell (Treg) differentiation, and facilitating immune tolerance during pregnancy (Fang et al., 2024; Jiang et al., 2021). Because HLA-G can be delivered to distant maternal immune cells through EVs, EV-mediated transport may extend the local immunosuppressive effects of trophoblasts beyond the immediate maternal–fetal interface (Alexandrova et al., 2025).

In addition to HLA-G, trophoblast-derived EVs contribute to immune privilege through the delivery of apoptosis-inducing ligands, including Fas ligand (FasL) and tumor necrosis factor-related apoptosis-inducing ligand (TRAIL). FasL-bearing EVs induce apoptosis of activated maternal Fas-positive T lymphocytes, whereas TRAIL selectively eliminates activated immune cells expressing death receptors while exerting minimal effects on resting lymphocytes. These complementary mechanisms prevent excessive maternal immune activation without causing generalized immunosuppression, thereby preserving immune homeostasis throughout pregnancy (Favaro et al., 2021; Morelli and Sadovsky, 2022). The coordinated expression of FasL and TRAIL within placental EVs therefore represents an important mechanism by which trophoblasts establish immune privilege during early pregnancy.

Another well-established mechanism involves EV-associated ligands for the activating receptor NKG2D. Placental EVs express multiple NKG2D ligands, including MICA, MICB, and ULBP family proteins, which bind to NKG2D receptors on maternal NK cells and cytotoxic CD8^+^ T cells. Sustained exposure to these EV-associated ligands promotes receptor internalization and downregulation, resulting in reduced cytotoxic activity of maternal immune cells. This mechanism limits immune-mediated damage to trophoblasts while maintaining an appropriate balance between fetal protection and maternal immune defense (Hedlund et al., 2009; Liu Y. et al., 2021). Together with HLA-G, FasL, and TRAIL, NKG2D ligand-bearing EVs constitute a multilayered immunoregulatory network that minimizes fetal antigen recognition by maternal immune cells.

Beyond membrane-associated proteins, EVs also transport numerous immunoregulatory microRNAs (miRNAs) that further shape immune cell function. Placental EVs are enriched in chromosome 19 microRNA cluster (C19MC) members, including miR-517a, miR-517b, miR-518b, and miR-519d, which have been implicated in antiviral defense and immune modulation. In addition, EV-associated miR-146a, miR-21, miR-155, and miR-223 regulate inflammatory signaling pathways, macrophage polarization, dendritic-cell maturation, and T-cell differentiation, thereby contributing to immune homeostasis at the maternal–fetal interface (Donker et al., 2012; Morelli and Sadovsky, 2022). Although the precise contribution of individual miRNAs remains incompletely understood, accumulating evidence suggests that EV-mediated transfer of regulatory miRNAs provides an additional layer of post-transcriptional control over maternal immune adaptation during pregnancy.

Collectively, EVs establish maternal immune tolerance through multiple complementary mechanisms, including inhibition of NK-cell activation, selective apoptosis of activated maternal lymphocytes, downregulation of immune-activating receptors, and delivery of immunoregulatory miRNAs. Rather than acting through a single signaling pathway, EVs integrate protein- and RNA-mediated communication to coordinate immune adaptation throughout pregnancy. Nevertheless, most mechanistic evidence is derived from *in vitro* studies or relatively small clinical cohorts, and future investigations employing standardized EV isolation methods, rigorous characterization according to MISEV recommendations, and *in vivo* functional validation are needed to define the specific contribution of individual EV cargoes to maternal immune tolerance.

### 3.2 EVs promote trophoblast adhesion and invasion

Successful embryo implantation relies on the precise regulation of trophoblast adhesion to the receptive endometrium, followed by controlled migration and invasion into the maternal decidua. These events require extensive remodeling of cell–cell and cell–extracellular matrix interactions and are coordinated through continuous communication between maternal and embryonic tissues. Increasing evidence indicates that EVs contribute to these processes by transferring bioactive molecules that regulate trophoblast motility, adhesion signaling, and extracellular matrix remodeling, thereby facilitating normal placental establishment.

Endometrial-derived EVs constitute an important component of the implantation microenvironment. Endometrial epithelial cells and stromal cells continuously secrete EVs into the uterine lumen, and their molecular composition is dynamically regulated by ovarian steroid hormones during the window of implantation. After uptake by trophoblasts or preimplantation embryos, these EVs influence the expression of genes involved in cytoskeletal organization, cell adhesion, migration, and extracellular matrix remodeling (Ma et al., 2022; Tan et al., 2021). Functional studies have demonstrated that EVs released from hormonally stimulated endometrial epithelial cells promote trophoblast attachment, migration, and spheroid outgrowth, suggesting that maternal EVs actively regulate embryo implantation rather than merely reflecting endometrial physiology (Greening et al., 2016; Jiang and Li, 2021).

Cell adhesion during implantation is largely mediated by integrins, which serve as key receptors linking trophoblasts to extracellular matrix components within the maternal decidua. Integrin heterodimers, including αVβ3, α5β1, and α6β4, are highly expressed in both trophoblasts and the receptive endometrium and are essential for blastocyst attachment, trophoblast migration, and invasion (Karimi et al., 2025; Nardo et al., 2002). Besides functioning as adhesion receptors, integrins also initiate intracellular signaling pathways that regulate cytoskeletal remodeling and focal adhesion dynamics. Recent studies in EV biology further suggest that EV-associated integrins contribute to selective cell targeting and intercellular communication, providing an additional mechanism by which EVs may coordinate interactions between trophoblasts and maternal tissues (Isogai et al., 2025). Although the specific functions of individual EV-associated integrins during embryo implantation remain to be fully elucidated, accumulating evidence supports an important role for integrin-dependent EV signaling in establishing endometrial receptivity.

Focal adhesion kinase (FAK) represents a major downstream effector of integrin signaling and plays a central role in trophoblast invasion. Upon integrin activation, FAK undergoes autophosphorylation and subsequently activates multiple signaling cascades, including the PI3K/AKT and MAPK/ERK pathways, thereby regulating focal adhesion turnover, actin cytoskeleton remodeling, and cell migration (Knofler, 2010; Liang et al., 2025). Although direct evidence demonstrating that endometrial EVs activate FAK signaling during implantation is still limited, several studies indicate that EV-mediated communication modulates integrin-dependent signaling networks involved in cell adhesion and motility. These observations suggest that FAK functions as an important signaling hub linking EV-mediated extracellular communication with trophoblast invasive behavior.

Collectively, current evidence indicates that EVs promote trophoblast adhesion and invasion through coordinated regulation of endometrial-derived signaling, integrin-mediated cell adhesion, and downstream FAK-dependent pathways. Rather than acting through isolated molecular mechanisms, these signaling networks integrate maternal and embryonic responses to establish a receptive implantation microenvironment. Nevertheless, the precise EV cargoes responsible for regulating trophoblast invasion remain incompletely defined, and most available evidence derives from *in vitro* models. Future studies employing standardized EV characterization, *in vivo* validation, and cell-specific functional analyses will be essential for elucidating the molecular mechanisms by which EVs regulate implantation.

### 3.3 EVs coordinate angiogenesis and vascular remodeling

Adequate placental angiogenesis and vascular remodeling are fundamental for establishing an efficient maternal–fetal circulation and sustaining fetal growth throughout pregnancy. These processes require tightly coordinated communication among trophoblasts, endothelial cells, decidual cells, and vascular smooth muscle cells. Recent studies have identified EVs as important mediators of this multicellular communication network, facilitating the transfer of angiogenic proteins, lipids, and regulatory RNAs that collectively regulate endothelial function, vascular remodeling, and placental vascular development (Beal et al., 2025; Ma et al., 2022).

One of the principal mechanisms by which EVs promote placental angiogenesis is through modulation of vascular endothelial growth factor (VEGF) signaling. Placenta-derived EVs contain numerous pro-angiogenic molecules, including VEGF, placental growth factor (PlGF), angiopoietins, and multiple angiogenesis-associated microRNAs. These cargoes stimulate endothelial cell proliferation, migration, survival, and capillary-like tube formation, thereby supporting the expansion and maturation of the placental vascular network (Cronqvist et al., 2017; Fatmous et al., 2022). In addition to directly transferring angiogenic mediators, EVs can alter the expression of VEGF signaling components in recipient endothelial cells, amplifying local angiogenic responses and facilitating vascular adaptation throughout gestation. Conversely, disruption of EV-mediated VEGF signaling has been associated with impaired placental vascularization and pregnancy complications such as preeclampsia and fetal growth restriction (Gebara et al., 2021; Morelli and Sadovsky, 2022).

Beyond VEGF signaling, EVs serve as important mediators of communication between trophoblasts and endothelial cells. Syncytiotrophoblast-derived EVs are readily internalized by maternal endothelial cells, where they modulate endothelial activation, nitric oxide production, inflammatory responses, and vascular permeability. These effects contribute to physiological vascular adaptation during pregnancy by maintaining endothelial integrity and promoting appropriate vascular remodeling (Gebara et al., 2021). In turn, endothelial-derived EVs can influence trophoblast proliferation, differentiation, and invasive capacity, suggesting that reciprocal EV exchange coordinates the development of both placental tissues and maternal vasculature. Such bidirectional communication enables maternal and fetal cells to dynamically adjust vascular function in response to the increasing metabolic demands of pregnancy (Ma et al., 2021).

Emerging evidence further indicates that EV-associated non-coding RNAs participate in fine-tuning angiogenic signaling networks. Placental EVs are enriched in several angiogenesis-related microRNAs, including miR-126, miR-210, and members of the chromosome 19 microRNA cluster (C19MC), which regulate endothelial cell survival, migration, and vascular remodeling by targeting components of the VEGF, PI3K/AKT, and hypoxia-inducible factor (HIF) signaling pathways (Xu et al., 2021; Zhang et al., 2021). Under physiological conditions, these EV-associated regulatory RNAs contribute to balanced vascular development. However, hypoxia, oxidative stress, or placental dysfunction can alter EV cargo composition, leading to endothelial dysfunction and aberrant angiogenesis that may contribute to pregnancy-related disorders (Fato et al., 2026; Hula et al., 2025).

Collectively, EVs coordinate placental angiogenesis through integrated regulation of angiogenic signaling pathways and reciprocal communication between trophoblasts and endothelial cells. By synchronizing vascular growth with placental development, EVs help establish a functional maternal–fetal circulation capable of supporting normal pregnancy. Nevertheless, the molecular mechanisms governing EV-mediated vascular remodeling remain incompletely understood, and considerable methodological heterogeneity among current studies—including differences in gestational age, EV isolation methods, and functional assays—limits direct comparison of findings. Future studies employing standardized EV characterization, longitudinal clinical sampling, and cell-specific functional analyses will be essential for defining the precise contribution of individual EV cargoes to placental angiogenesis.

### 3.4 EVs mediate epithelial–stromal crosstalk

Successful pregnancy requires continuous communication between maternal endometrial cells and fetal trophoblasts to coordinate tissue remodeling and maintain the structural and functional integrity of the maternal–fetal interface. Rather than acting through isolated signaling events, EVs facilitate reciprocal communication among endometrial epithelial cells, stromal cells, decidual cells, and trophoblasts by transferring proteins, lipids, and regulatory RNAs. This bidirectional EV-mediated signaling enables maternal and fetal tissues to adapt dynamically to the changing physiological demands of early pregnancy and supports the establishment of a stable implantation microenvironment (Beal et al., 2023).

Accumulating evidence indicates that EVs derived from endometrial epithelial cells regulate stromal cell function during decidualization while simultaneously influencing trophoblast behavior. Hormonal stimulation by estrogen and progesterone alters the molecular composition of endometrial EVs, enabling the transfer of signaling molecules that regulate cell proliferation, differentiation, extracellular matrix organization, and cytokine secretion (Fatmous et al., 2022; Komsky-Elbaz et al., 2025). These coordinated responses promote the transformation of endometrial stromal fibroblasts into specialized decidual cells while enhancing the capacity of trophoblasts to adapt to the maternal microenvironment. Thus, EVs contribute not only to embryo implantation but also to the establishment of a supportive decidual niche that sustains early placental development.

Reciprocal signaling from trophoblasts to maternal tissues represents an equally important component of epithelial–stromal crosstalk. Trophoblast-derived EVs are internalized by endometrial epithelial cells, stromal cells, and decidual immune cells, where they modulate the expression of genes associated with extracellular matrix remodeling, cytokine production, cellular differentiation, and tissue homeostasis (Beal et al., 2023; Komsky-Elbaz et al., 2025). Through these mechanisms, trophoblast-derived EVs actively reshape the maternal decidual environment, enabling synchronized development of fetal and maternal tissues while preventing excessive tissue injury during controlled trophoblast invasion.

In addition to mediating communication between maternal and fetal cells, EVs coordinate interactions among different maternal cell populations within the decidua. Emerging evidence suggests that EV-associated regulatory RNAs and proteins influence matrix metalloproteinases (MMPs), tissue inhibitors of metalloproteinases (TIMPs), transforming growth factor-β (TGF-β), and other signaling molecules involved in extracellular matrix turnover and tissue remodeling (Nabeel et al., 2026; Wu et al., 2026). These signaling networks maintain the balance between trophoblast invasion and decidual integrity, ensuring that placental development proceeds without excessive disruption of maternal tissues.

Collectively, EV-mediated epithelial–stromal crosstalk represents a dynamic communication network that coordinates maternal tissue remodeling with fetal development throughout early pregnancy. Unlike the EV-mediated regulation of immune tolerance, trophoblast invasion, or angiogenesis discussed above, this mechanism emphasizes reciprocal communication among multiple maternal and fetal cell types to maintain tissue homeostasis at the maternal–fetal interface. Future studies integrating spatial transcriptomics, single-cell multiomics, and standardized EV characterization will provide greater insight into the spatiotemporal dynamics of EV-mediated epithelial–stromal communication during normal and complicated pregnancies.

### 3.5 EVs regulate metabolic and inflammatory adaptation

Pregnancy is characterized by profound metabolic and immunological adaptations that enable the maternal–fetal interface to meet the increasing demands of fetal growth while maintaining tissue homeostasis. Rather than functioning solely as mediators of intercellular communication, EVs actively coordinate these adaptive responses by transferring bioactive lipids, proteins, and regulatory RNAs between maternal and fetal cells. Through continuous modulation of metabolic activity and inflammatory signaling, EVs help establish a balanced microenvironment that supports placental function and normal pregnancy progression (Lin et al., 2026).

Emerging evidence suggests that EV-associated lipids play important regulatory roles in metabolic adaptation during pregnancy. Among these, glycosphingolipids have attracted increasing attention because they participate in membrane organization, signal transduction, and cell–cell interactions. Glycosphingolipids carried by placental EVs contribute to trophoblast differentiation, membrane stability, and intracellular signaling, while also influencing maternal immune cell responses and metabolic homeostasis (Sheng et al., 2025; Zhang et al., 2025). Alterations in EV lipid composition under pathological conditions, including hypoxia and oxidative stress, may disrupt cellular metabolism and impair placental function, suggesting that lipid cargoes constitute an important component of EV-mediated maternal–fetal communication (Zhang et al., 2023).

In addition to lipid cargoes, EV-associated microRNAs participate in regulating maternal metabolic adaptation by modulating gene expression in recipient cells. Placental EVs contain numerous metabolism-related microRNAs, including members of the chromosome 19 microRNA cluster (C19MC), as well as miR-16, miR-21, miR-29, miR-122, and miR-210, which regulate glucose metabolism, insulin signaling, mitochondrial function, and cellular responses to hypoxia (Fu et al., 2024; Morales-Prieto et al., 2020). Through post-transcriptional regulation of metabolic pathways, these EV-associated microRNAs facilitate physiological adaptation of maternal tissues to pregnancy while supporting placental growth and fetal development. Dysregulation of these metabolic miRNAs has been associated with gestational diabetes mellitus and fetal growth restriction, highlighting their importance in maintaining metabolic homeostasis (Szuszkiewicz et al., 2022).

EVs also serve as important modulators of inflammatory adaptation throughout pregnancy. Placental EVs carry diverse cytokines, chemokines, damage-associated molecular patterns, and immunoregulatory microRNAs that collectively regulate inflammatory responses at the maternal–fetal interface (Lewis et al., 2025; Morelli and Sadovsky, 2022). Under physiological conditions, EV-mediated signaling contributes to the establishment of a controlled inflammatory environment that supports trophoblast function, vascular remodeling, and tissue repair (Yanez-Mo et al., 2015). However, environmental stressors such as infection, oxidative stress, or placental hypoxia can alter the molecular composition of EVs, resulting in excessive production of pro-inflammatory mediators and disruption of maternal–fetal homeostasis. Such alterations are increasingly recognized as important contributors to pregnancy complications, including preeclampsia, preterm birth, and gestational diabetes mellitus (Lewis et al., 2025).

Collectively, EVs integrate metabolic and inflammatory signaling to coordinate physiological adaptation at the maternal–fetal interface. By simultaneously regulating lipid metabolism, gene expression, and inflammatory responses, EVs enable maternal and fetal tissues to respond dynamically to the changing physiological demands of pregnancy. Importantly, disruption of these adaptive mechanisms may represent a common pathogenic pathway underlying multiple pregnancy-related disorders. A better understanding of EV-mediated metabolic and inflammatory regulation will therefore not only improve our knowledge of normal pregnancy biology but also provide a mechanistic framework for interpreting EV dysregulation in pregnancy complications discussed in the following section.

## 4 Role of EVs in pregnancy-related diseases

EVs have emerged as pivotal mediators of pregnancy-associated pathologies, with growing evidence highlighting their role in the pathogenesis and potential early detection of disorders such as preeclampsia (PE), gestational diabetes mellitus (GDM), preterm birth (PTB), recurrent implantation failure (RIF), and fetal growth restriction (FGR). Table 1 summarizes representative studies and key molecular findings related to these pregnancy-associated complications.

**TABLE 1:** Roles of EVs in pregnancy-related disorders.

Disease	Source of EVs	EV cargo	Functions/Effects	Methods	References
PE	Placenta-derived exosomes (pEXOs)	miR-519d-3p	Disrupts maternal immune tolerance by enhancing T cell proliferation, survival, and Th17 skewing	miRNA-seq, WB, RT-qPCR, CCK-8 assay, apoptosis assay, *in vitro* co-culture of HTR-8/SVneo and Jurkat T cells	Cao et al. (2025)
PE	Syncytiotrophoblast-derived EVs (STEVs)	HLA-DR	Elevated HLA-DR^+^ STEVs associated with PE onset	Plasma EV isolation by ultracentrifugation, WB, nanoparticle tracking, flow cytometry for PLAP and HLA-DR	Onori et al. (2024)
PE	Placenta, maternal blood	miRNAs (19MC, 14 MC clusters)	Predict late-onset PE (AUC = 0.96); regulate vascular remodeling, growth, and VEGF/EGF–PIP3/Akt signaling	miRNA seq	Ghosh et al. (2024)
PE	Trophoblast-derived EVs (PE vs. NP)	Altered lipids (∼37 species)	Promote M1 polarization and systemic inflammation; trigger PE-like symptoms in mice, alleviated by macrophage depletion	Flow cytometry, immunochemistry, TEM, NTA, qRT-PCR, lipidomics-transcriptomics, mouse model	Liu et al. (2022)
PE	HTR8-/Svneo cells and macrophages	miR-141–3p	Inhibits trophoblast invasion and mitophagy; promotes M1 macrophage polarization and impairs spiral artery remodeling	*In vitro* culture, co-culture, mouse model, molecular analyses	Wu et al. (2024)
PE	Maternal plasma	Proteins (e.g., PlGF, PTX3, VEGFR-1, CCL3, endoglin)	Identify distinct PE subtypes; enable early prediction (AUC: preterm 78%, term 68%)	multiplexed immunoassays, statistical modeling	Than et al. (2024)
PE	Amniotic fluid	CD105 (endoglin)-enriched EVs	Exert anti-angiogenic effects (endoglin decoy), reflecting a hypoxic and anti-angiogenic environment	Bead-based cytofluorimetry, chip-based analysis, *in vitro* functional assays	Gebara et al. (2022)
PE	Medium/Large STB-EVs (placenta)	miRNAs (e.g., hsa-miR-193b-5p, 324–5p, 652–3p, 3196, 9–5p, 421, 210–3p)	Altered miRNAs link placental stress to PE pathogenesis; hsa-miR-9-5p as potential biomarker	*Ex vivo* perfusion, small RNA-seq, qPCR, bioinformatics	Awoyemi et al. (2024)
PE	Plasma placental EVs (pcEVs)	Anionic phospholipids, VWF	Elevated pcEVs (and other EVs) associated with hypercoagulability and PE prediction	Flow cytometry, coagulation assays, ELISA	Chen et al. (2021)
PE	Syncytiotrophoblast-derived EVs (microvesicles, exosomes)	Neprilysin (NEP)	Elevated NEP activity on EVs promotes hypertension and may contribute to PE pathogenesis	Immunostaining, Western blot, magnetic bead isolation, flow cytometry, SEC	Gill et al. (2019)
PE	Syncytiotrophoblast-derived EVs (microvesicles, exosomes)	eNOS (endothelial NO synthase)	Reduced eNOS-derived NO activity in PE EVs may contribute to decreased NO bioavailability and vascular resistance	*Ex vivo* placental perfusion, Western blot, flow cytometry, immunodepletion, NO activity assays	Motta-Mejia et al. (2017)
GDM	Plasma sEVs (normal pregnancy vs. GDM)	Unspecified bioactive cargo	sEVs from healthy pregnancy enhance GSIS and insulin responsiveness; GDM sEVs impair GSIS and exacerbate insulin resistance	Mouse infusion, glucose tolerance test, islet and muscle analyses	James-Allan et al. (2020)
GDM	Maternal plasma EVs	miRNAs (e.g., miR-92a-3p)	Altered EV-miRNAs across gestation enable early GDM prediction; miR-92a-3p promotes skeletal muscle insulin sensitivity via JAK-STAT signaling	Small RNA-seq, qPCR, DIA-MS, functional *in vitro* assays	Nair et al. (2021)
GDM	STBEVs (medium/large vs. small)	Proteins (56 differential in m/lSTBEVs)	Altered protein profiles affect cytoskeleton organization and maternal-fetal signaling	*Ex vivo* placental perfusion, MS-based proteomics, bioinformatics	Kandzija et al. (2024)
GDM	Maternal plasma EVs (placental origin)	miRNAs (19MC, 14MC, miR-92a-3p, 192–5p, 451a, 122–5p, 100–5p, 125a-3p)	Early prediction (AUC 0.95–0.96) of GDM by EV-miRNAs targeting genes in PI3K, FOXO, insulin, and glucogenic pathways	Longitudinal EV isolation, RNA-seq, logistic regression, LOOCV	Thamotharan et al. (2022)
PTB	Maternal plasma exosomes	miRNAs (∼173 differentially expressed across gestation)	Reflect gestation progression and differences between term and preterm birth via TGF-β, p53, and glucocorticoid signaling	Longitudinal EV isolation, small RNA-seq, bioinformatic pathway analysis	Menon et al. (2019)
PTB	Cord plasma exosomes	Proteins	Similar quantity and markers across conditions; proteome lacks specificity as a biomarker for parturition	Differential centrifugation, SEC, NTA, dot blot, LC-MS/MS, IPA	Menon et al. (2022)
PTB	Maternal plasma exosomes (E18, mouse	Inflammatory mediators	Exosomes from late gestation (E18) induce preterm birth via localized inflammatory responses	Exosome isolation, characterization, *in vivo* injection, immunohistochemistry	Sheller-Miller et al. (2019)
PTB	Amnion epithelial cell-derived exosomes (eHMGB1)	HMGB1 protein	Exosomes carry HMGB1, propagate across fetal-maternal interface, trigger inflammation and preterm birth	Organ-on-a-chip, exosome engineering, *in vivo* mouse injections, immunohistochemistry	Radnaa et al. (2021)
PTB	First-trimester urine-derived EVs	Cytokines (MCP-1, IL-6, IL-17A, IP-10, TNFα, IL-12, IFNγ)	Reflect altered M1 and Th17 differentiation, predictive of PB, and modulate trained immunity	EV isolation, immunoassays, qPCR, histone modification (H3K4me3) analyses	Huang et al. (2023)
PTB	Maternal plasma EVs-exosomes	sFLT-1, PlGF	Altered sFLT-1/PlGF ratio in EVs-EXs is associated with PTB risk and may serve as a predictive biomarker	ELISA of maternal plasma and EV-EXs, statistical analyses	Hussein et al. (2022)
PTB	HEK293T-derived EVs	eIL-10	Reduce maternal-fetal inflammation, NF-κB activation, and pro-inflammatory cytokine production, delay PTB	Electroporation of EVs, *in vitro* macrophage LPS challenge, *in vivo* mouse model with *E. coli*, CyTOF analysis	Kammala et al. (2023)
PTB	PLAP^+^ placental EVs	Proteins (96 diff.)	Altered protein profiles across gestation. Upregulation of inflammatory and EMT pathways at term, downregulation of coagulation/complement in PTB. Potential for early PTB biomarker development	Longitudinal maternal plasma collection across trimesters, PLAP-based enrichment, SWATH MS proteomics, bioinformatics analysis	Menon et al. (2020)
RIF	Endometrial cells from RIF patients and fertile women (cultured and hormonally treated)	Classic EV markers: TSG101, Alix, CD9 (protein markers)	RIF-EVs reduce embryo development: decrease blastocyst rate, hatching rate, total cell number, and invasion capacity compared to fertile EVs	Isolation by ultracentrifugation; characterization by Western blot, TEM, NTA; fluorescent labeling for EV uptake; co-culture with 2-cell murine embryos; blastocyst and hatching rate calculation; DAPI staining for cell count; invasion assay on Ishikawa cells	Liu et al. (2020)
RIF	Endometrial cells from RIF patients and fertile women (hormonally modulated)	Differentially expressed miRNAs (11 identified; 4 validated, notably miR-6131)	Altered miRNAs in RIF-EVs may impair embryo implantation by inhibiting trophoblast cell growth and invasion (e.g., miR-6131 inhibits HTR8/SVneo cell growth and invasion)	EV isolation by ultracentrifugation; characterization by Western blot, NTA, TEM; miRNA sequencing; qRT-PCR validation; target gene prediction; functional assay on trophoblast cell line (HTR8/SVneo)	Liu et al. (2021a)
RIF	Endometrial EVs from RIF patients; engineered EVs (E-EVs) from human umbilical cord mesenchymal stem cells (HUMSCs)	miR-218–5p and anti-miR-218–5p	miR-218–5p inhibits mouse embryo development, reducing blastocyst and hatching rates, impairing trophectoderm (TE) and ICM gene expression; anti-miR-218–5p in engineered EVs reverses these effects	miR-218–5p agomir treatment in mouse embryos; blastocyst/hatching rate assessment; RNA-seq; qRT-PCR; immunofluorescence; Western blot; NTA; TEM; EV internalization visualization by BODIPY TR ceramide staining	Cai et al. (2025)
FGR	Placenta/trophoblast exosomes	PPARγ protein	Exosomes deliver PPARγ to preadipocytes, promoting adipogenesis and rescuing FGR-associated adipogenic defects	Mouse trophoblast-specific PPARγ ablation, trophoblast–preadipocyte coculture, exosome isolation, RGZ nanoparticle delivery, adipogenic gene analysis	Luo et al. (2025)
FGR	Maternal and fetal plasma exosomes (placenta-derived)	Placental markers (PLAP), exosomal proteins (CD63)	Placental exosome ratio correlates with fetal growth; decreased placental exosome contribution in FGR reflects impaired placental function	Plasma exosome isolation, characterization, and quantification using EM, WB, NTA, and quantum dot labeling; statistical correlation analysis	Miranda et al. (2018)

### 4.1 Preeclampsia

PE is a multifactorial pregnancy disorder associated with placental dysfunction and maternal–fetal immune imbalance. Increasing evidence highlights placental EVs as key mediators and potential biomarkers in its pathogenesis. Placenta-derived EVs carry distinct miRNA profiles that modulate angiogenesis and immune tolerance. Ghosh et al. (2024) identified a C19MC/C14 MC miRNA signature in maternal EVs that predicted late-onset PE with high accuracy (AUC = 0.96), implicating VEGF, EGF, and PI3K/Akt signaling (Ghosh et al., 2024). MiR-519d-3p, a highly expressed C19MC member, was significantly elevated in PE-EVs and shown to promote T cell proliferation, inhibit apoptosis, and induce Th17 polarization, thereby disrupting Th17/Treg balance and maternal immune tolerance (Cao et al., 2025). Other EV-contained miRNAs, such as hypoxia-induced miR-141–3p, can polarize macrophages toward a pro-inflammatory M1 phenotype via the TAB2/TAK1 pathway, impairing trophoblast invasion (Wu et al., 2024). Similarly, altered lipid and transcriptomic profiles in PE-EVs were linked to macrophage-driven inflammation and immune dysregulation (Liu et al., 2022).

Beyond miRNAs, protein cargo in EVs also reflects disease status. Than et al. (2024) identified altered angiogenic (*PlGF*, *VEGFR-1*) and inflammatory (*PTX3*, *CD40L*) markers in PE-EVs (Than et al., 2024). HLA-DR^+^ syncytiotrophoblast-derived EVs (STEVs), elevated throughout gestation in PE patients, may reflect aberrant immune phenotypes and offer early diagnostic value (Onori et al., 2024). Additional studies have linked PE-EVs to vascular dysfunction, showing increased neprilysin (NEP) activity (Gill et al., 2019) and reduced eNOS levels (Motta-Mejia et al., 2017). Circulating miRNAs such as miR-9-5p (Awoyemi et al., 2024), together with changes in EV-associated *sFLT-1* and *PlGF* (Chen et al., 2021; Hussein et al., 2022), further support their potential as early biomarkers. Amniotic fluid-derived CD105^+^ EVs with anti-angiogenic activity also appear elevated in PE (Gebara et al., 2022). Together, these findings position placental EVs as active contributors to PE pathophysiology and promising tools for early diagnosis.

### 4.2 Gestational diabetes mellitus

EVs have also been implicated in maternal glucose metabolism and GDM pathogenesis. James-Allan et al. showed that EVs from healthy pregnancies enhance glucose-stimulated insulin secretion and foster physiological insulin resistance, whereas GDM-derived EVs impair insulin signaling and glucose tolerance (James-Allan et al., 2020). Nair et al. identified over 500 differentially expressed EV-miRNAs across pregnancy, with a panel achieving >90% diagnostic accuracy for GDM. Notably, EV-derived miR-92a-3p was found to target the JAK–STAT pathway and regulate maternal insulin sensitivity (Nair et al., 2021). Similar findings by Thamotharan et al. revealed placental EV-miRNAs (e.g., miR-92a-3p, miR-192–5p) as early predictive markers for GDM, acting through the PI3K, FOXO, and insulin signaling pathways (Thamotharan et al., 2022). Meanwhile, Kandzija et al. demonstrated distinct proteomic profiles of placental EVs in GDM, suggesting that specific protein cargoes may disrupt maternal–fetal metabolic signaling (Kandzija et al., 2024).

### 4.3 Preterm birth

In preterm birth and parturition, EVs have been implicated in mediating inflammatory and immunological shifts that trigger labor. Menon et al. identified gestation-dependent exosome-miRNA profiles associated with term versus preterm delivery, highlighting the role of TGF-β, p53, and glucocorticoid signaling (Menon et al., 2019). Similarly, Sheller-Miller et al. demonstrated that late-gestation maternal exosomes can precipitate preterm birth by inducing NF-κB-dependent inflammatory cascades upon administration to pregnant mice (Sheller-Miller et al., 2019). Radnaa et al. revealed that exosomes carrying HMGB1 from senescent amniotic epithelial cells can activate RAGE and TLR4 pathways, triggering preterm parturition (Radnaa et al., 2021). Huang et al. identified urinary EV–mRNA and cytokine profiles that reliably distinguished preterm from term birth, suggesting a role for early immunomodulatory signaling (Huang et al., 2023). Hussein et al. reported altered sFLT-1 and PlGF in EV-exosomes associated with preterm deliveries, providing potential diagnostic markers (Hussein et al., 2022). Meanwhile, Kammala et al. demonstrated that engineering EVs for targeted delivery of IL-10 can ameliorate infection-induced preterm birth, highlighting therapeutic opportunities (Kammala et al., 2023).

### 4.4 Recurrent implantation failure

EVs from the endometrium have been implicated in implantation outcomes. Liu et al. demonstrated that EVs from patients with RIF disrupt mouse embryonic development and invasiveness, suggesting a role for abnormal EV-derived cargo (Liu et al., 2020). Further analyses identified dysregulated miRNAs, including miR-6131 and miR-218–5p, as key mediators of implantation failure. Importantly, delivery of anti-miR-218–5p via engineered EVs promoted trophectoderm and inner cell mass differentiation and improved blastocyst development, presenting a promising therapeutic avenue (Cai et al., 2025; Liu C. et al., 2021).

### 4.5 Fetal growth restriction

Placenta-derived EVs have also been implicated in fetal growth and adipose tissue development. Luo et al. uncovered placental PPARγ as a critical mediator of fetal adipogenesis via exosome-derived signaling, suggesting that its downregulation contributes to FGR pathogenesis (Luo et al., 2025). In clinical studies, Miranda et al. identified altered placental EV proportions (PLAP+/CD63+) in maternal and fetal circulation as highly associated with FGR and placentation quality, supporting the role of placental EV profiles as sensitive indicators of placentation and fetal growth status (Miranda et al., 2018).

Cumulative evidence highlights the central role of EV-derived cargoes in regulating maternal–fetal interactions and their profound impact across pregnancy-related disorders. By mediating cellular communication, immune modulation, and placentation, EVs serve both as mechanistic drivers and as promising biomarkers for early prediction and therapeutic intervention in pregnancy complications.

## 5 Clinical applications and technical challenges

EVs play a pivotal role in regulating interactions at the maternal-fetal interface, offering significant clinical application potential, particularly in the early diagnosis, treatment, and prognostic evaluation of pregnancy-related diseases (Liu et al., 2024; Zhou et al., 2020). Their unique properties, such as stability, efficient intercellular transport, and ease of detection in bodily fluids, position EVs as ideal candidates for non-invasive biomarkers and therapeutic tools (Ciferri et al., 2021; Erdbrugger et al., 2021). However, substantial methodological, technical, and translational challenges remain before EV-based approaches can be routinely implemented in clinical practice.

The potential of EVs as tools for early diagnosis has garnered significant attention. Due to their presence in maternal circulation from the early stages of pregnancy, the miRNA, proteins, and other biomolecules carried by EVs can reflect the physiological status of the maternal-fetal interface (Foley et al., 2021). For instance, specific placental-derived EV-associated molecules have been reported to be associated with pregnancy complications such as preeclampsia, preterm birth, and fetal growth restriction (Ortega et al., 2022). Consequently, EV-based liquid biopsy represents a promising strategy for non-invasive biomarker discovery and early risk assessment of pregnancy complications (Chaemsaithong et al., 2023). However, most reported biomarkers remain at the discovery stage and require validation in independent, large-scale prospective cohorts before clinical implementation.

The therapeutic potential of EVs has also attracted increasing interest. Beyond serving as diagnostic biomarkers, EVs can be engineered or utilized as drug carriers because of their ability to target specific cell types, including placental cells and maternal immune cells. This targeting capability enables the delivery of therapeutic molecules, such as anti-inflammatory agents, miRNAs, or siRNAs, directly to diseased tissues while potentially reducing systemic side effects (Kooijmans et al., 2021; Uddin et al., 2024). Such approaches may provide novel strategies for modulating maternal immune responses or restoring placental function in pregnancy-related disorders (Condrat et al., 2021). Despite the considerable interest in EV-based therapeutics, their translation into maternal–fetal medicine remains challenging. Unlike many other clinical settings, pregnancy requires simultaneous consideration of maternal efficacy and fetal safety. Critical issues remain unresolved, including biodistribution, placental transfer, fetal exposure, immunogenicity, dose optimization, cargo stability, off-target effects, large-scale manufacturing, quality control, and regulatory approval. Consequently, most EV-based therapeutic strategies remain in the preclinical stage, and substantial safety and efficacy studies are required before clinical implementation can be considered.

Despite the immense potential of EVs in diagnostics and therapeutics, their clinical application faces a series of technical challenges. First, the isolation and purification techniques for EVs require further optimization (Liangsupree et al., 2021). Current methods, such as differential centrifugation, ultracentrifugation, and immunocapture, are often complex, yield low purity, and provide limited quantities, which hampers large-scale applications (Sidhom et al., 2020). Second, the *in vivo* stability, distribution, and targeting of EVs remain incompletely understood, further complicating their reproducibility and clinical application (Cabral et al., 2024). Furthermore, the intrinsic heterogeneity of EV populations presents substantial challenges for standardization and quality control (Wakker et al., 2023). Given that EVs secreted under different physiological conditions and from different cellular sources differ considerably in their molecular composition and biological functions, establishing standardized isolation, characterization, and analytical protocols remains an urgent priority (Mohammadipoor et al., 2023). Moreover, despite the growing number of studies investigating EVs in pregnancy, substantial methodological heterogeneity remains a major limitation. Differences in sample collection, gestational age, isolation procedures, EV characterization, normalization methods, and analytical platforms complicate cross-study comparisons. In addition, many proposed EV biomarkers have been identified in relatively small cohorts and lack independent external validation. Future studies should adopt standardized protocols, adhere to the MISEV recommendations, incorporate longitudinal study designs, and account for important biological confounders such as maternal BMI, parity, fetal sex, medication exposure, and disease severity.

Addressing these methodological and technical limitations will be essential for the successful clinical translation of EVs. Future research should therefore focus on several key areas. First, more efficient and standardized methods for EV isolation and purification are needed to improve yield, purity, and reproducibility (Sanz-Ros et al., 2023). Second, engineering strategies should be further optimized to enhance the targeting specificity, therapeutic efficacy, and safety of EV-based delivery systems (Fei et al., 2024). Additionally, a deeper understanding of EV biology and their dynamic regulatory mechanisms at the maternal–fetal interface is required to facilitate the development of disease-specific diagnostic and therapeutic strategies (Guzewska et al., 2023). Furthermore, advances in nanotechnology and bioengineering may enable the development of hybrid delivery systems combining synthetic nanoparticles with natural EVs, thereby expanding the therapeutic potential of EV-based platforms (Ezike et al., 2023).

Overall, EVs exhibit significant clinical application potential in pregnancy-related diseases; however, their translation into routine clinical practice remains limited by methodological, technical, and safety challenges. Continued advances in EV biology, standardized methodologies, bioengineering, and clinical validation are expected to accelerate the development of reliable EV-based diagnostic and therapeutic strategies for pregnancy-related disorders.

## 6 Conclusion and future perspectives

EVs as key regulators of signaling between the mother and fetus, have increasingly become a significant focus in pregnancy biology research. This review explores the biogenesis, molecular mechanisms, and roles of EVs at the maternal-fetal interface, delving into how they regulate the complex signaling networks that maintain pregnancy homeostasis. The selective molecular packaging mechanism, targeted delivery capabilities, and environmental responsiveness of EVs are critical in mediating maternal-fetal immune regulation, sustaining placental function, and influencing the development of pregnancy-related diseases (Kumar et al., 2024; Mincheva-Nilsson and Baranov, 2014).

Nonetheless, the clinical translation of EVs faces numerous technical challenges. Key issues that require urgent resolution include optimizing the isolation and purification techniques for EVs, enhancing their targeted delivery efficiency, mitigating potential immunological risks, and ensuring their stability and safety within the body (Di Bella, 2022; Ramirez et al., 2018). Additionally, the biological heterogeneity and complexity of the molecular functions of EVs necessitate further exploration of their mechanisms, particularly regarding the specific roles of EVs derived from different sources and types in the maternal-fetal interface and pregnancy-related diseases (Buca et al., 2020).

Overall, EVs have emerged as key mediators of maternal–fetal communication and represent promising candidates for biomarker discovery and therapeutic development. However, further mechanistic studies, methodological standardization, and rigorous clinical validation are essential before EV-based approaches can be translated into routine clinical practice. Continued advances in nanotechnology, bioengineering, molecular diagnostics, and reproductive biology are expected to accelerate the safe and effective clinical application of EVs, ultimately improving the diagnosis, prevention, and management of pregnancy-related disorders.
